# Food-Borne Parasitic Zoonoses: Fish and Plant-Borne Parasites (World Class Parasites)

**DOI:** 10.3201/eid1409.080495

**Published:** 2008-09

**Authors:** Frank J. Sorvillo

**Affiliations:** Author affiliation: University of California, Los Angeles, California, USA

With >4,000 biomedical journals currently available, and more being added seemingly every month, one of the most overwhelming and daunting tasks for any health professional is to stay current with the literature. A welcomed lifeline has been the World Class Parasites series of publications that have made this challenge much easier to manage. The latest text (Volume 11), Food-Borne Parasitic Zoonoses: Fish and Plant-Borne Parasites, is a superb addition to this series. Murrell and Fried have brought together a stellar group of contributors in a comprehensive and up-to-date review of the helminthic zoonoses that are transmitted through fish and plant consumption. Given that the distribution and effect of these zoonoses remain woefully underappreciated, they are deserving of the justifiable attention brought by this book.

The first 9 chapters provide a thorough discussion of intestinal, liver, and lung flukes; fish-borne tapeworms; and tissue nematodes. Two superb chapters on immunologic aspects and molecular epidemiology of these parasites complete the text. There is much to extol in this volume. In an era of justifiable focus on molecular biology and genetics, it is heartening to see the broad-based and integrated approach taken by the editors and collaborators. This approach successfully accomplishes the stated objective of the editors to “… celebrate the diversity of approach that comprises modern parasitological research.” The chapters are clearly written and well organized with a balance of both classic, historic articles as well as important recent work.

The editors and authors have obviously made a priority of ensuring that the material is widely accessible to scientists and health professionals in diverse disciplines. They have presented detailed information on the biology, life cycles, natural history, diagnosis, treatment, epidemiology, and control of the agents or infections discussed. Current diagnostic techniques are thoroughly addressed. Insightful discussion is provided at the end of each chapter on current gaps in knowledge and research needs that offers a coherent roadmap for future studies. The chapter on intestinal capillariasis by Cross and Belizario is particularly noteworthy. Abundant images of exceptional quality are included and this adds substantially to the text. More color images would have been of value, but they may have made the text prohibitively expensive. Some chapters could have had additional discussion of epidemiologic aspects and a few typos can be found, but these do not diminish the value of this outstanding work.

Food-Borne Parasitic Zoonoses: Fish and Plant-Borne Parasites is a text of exceptional quality that should have broad appeal and utility for health professionals, researchers, medical and public health students, and policy makers. Given the increasing interest in neglected tropical diseases, this book is a timely and commendable work that will promote efforts to reduce the effects of these parasitic agents.

